# Grand Challenges in Oral Surgery

**DOI:** 10.3389/froh.2020.00005

**Published:** 2020-06-18

**Authors:** Adalberto L. Rosa, Emanuela P. Ferraz

**Affiliations:** ^1^Department of Oral & Maxillofacial Surgery and Periodontology, School of Dentistry of Ribeirão Preto, University of São Paulo, São Paulo, Brazil; ^2^Department of Maxillofacial Surgery, Prosthodontics and Traumatology, School of Dentistry, University of São Paulo, São Paulo, Brazil

**Keywords:** oral surgery, third molar/surgery, internal fixation, regenerative medicine, virtual surgical planning, robotic surgery

“Oral surgery” was a term coined by James E. Garretson in the middle of the nineteenth century [1] that nowadays encompasses a myriad of procedures that are within the scope of this section. From the Edwin Smith Papyrus, said to be from around 1700 BC but probably dating back much further to 3000 BC, to the writings of Hippocrates and Aristotle reporting on dental extraction to maxillomandibular wire fixation between 300 and 500 BC, to the books by Ambroise Paré, Richard Wiseman, and Pierre Fauchard in the sixteenth and eighteenth centuries [2], knowledge in the oral surgery field has been mainly focused on both improving the patient outcome and minimizing morbidity. At least in part, this advancement results from the fact that oral surgery has embraced findings from distinct areas such as cell biology, biomaterials, physics, and engineering.

The most common oral surgery procedure is the removal of the third molar. Despite development of surgical techniques related to the flap design, osteotomies, and pre- and post-operative care, lower third removal mainly results in post-operative morbidities such as pain, edema, and trismus. Many papers have been published reporting on such issues as the effect of drugs, the combinations of drugs with different mechanisms of action, comparing pre-operative vs. post-operative administration of drugs, laser, acupuncture, cryotherapy, etc., but there still are some improvements to be made in this area. Controversies regarding the preventive or prophylactic removal of unerupted and asymptomatic third molars have been discussed for decades, still without consensus. Some authors opt for the prophylactic tooth extraction, assuming that it reduces surgical complications due to: the early age of the patient, the prevention of the development of cysts and tumors, the minimizing of infection risk, the avoidance of orthodontic disturbances such as anterior dental crowding, and the reducing of periodontal damage to the second molars, among other factors. However, a recent systematic review showed a lack of scientific evidence, and high-quality research is urgently needed to support the prophylactic removal of third molars [3].

The evolution of internal fixation principles and devices were perhaps the most impactful developments in oral surgery. By joining the concepts of histology, anatomy, biomechanics, metallurgy, and polymer science, techniques and biomaterials have been used to treat facial fractures and deformities. While the use of plates to treat orthopedic fractures dates back to the early twentieth century, a major development in this area occurred after World War II [4] and, not surprisingly, it parallels the increase in knowledge of bone biology and biomaterials. As recently reviewed by Yeoh and Cunnigham [5], firstly, oral surgery borrowed devices from orthopedics, using plates to treat fractures of extremities that had proven to be successful [6, 7], and which was very soon followed by the development of devices, plates, and screws suitable for oral surgery procedures [8]. Also, the benefits of internal fixation in orthognathic surgery have been recently reviewed by Perez and Liddell [9] and Bell [10], and the formers raised questions that remain to be answered concerning hardware designs, types, and amount, as well as techniques and planning. Originally, internal fixation systems were metallics, made from stainless steel, cobalt-chromium-molybdenum alloy, and titanium and some of its alloys. The use of titanium reduced some of the concerns with these devices, however, many other issues, mainly based on the fact that they are permanent, remain. So, the lack of a perfect adaptation, deleterious effects on cranial growth, thermal sensitivity, palpations, titanium particles' migration to distant areas, and other issues have led to the need for a second surgical intervention to remove these systems that have not been solved by their miniaturization. In the late 1980s, polymer-based devices were introduced in part to solve the problems related to the use of metallic devices. However, these also presented some drawbacks including inflammation, body foreign reaction, and unpredictable resorption. The debate between metallic vs. polymeric systems is still present. The customization of these devices based on virtual planning and 3D printing and the incorporation of bioactive molecules to be delivered to the fractures and osteotomies sites seem to be a step forward.

Oral surgery plays an important role in the repair/regeneration of tissue loss in the craniofacial area, notably bone, resulting from traumatic injuries, degenerative or congenital diseases, and aging. Autogenous grafts are the gold standard treatment, due to their osteogenic, osteoconductive, and osteoinductive properties. However, their drawbacks include an increased morbidity and a limited available amount. Distraction osteogenesis has been successfully applied for the treatment of several conditions from the recovery of alveolar ridge height to major facial deformities, but it is not free of complications either. The increasing clinical demand for replacing or regenerating cells, tissue, or organs, aiming to restore or establish function, have driven the search for the development of new strategies and adjunctive therapies since the 1930's [11]. In this context, regenerative medicine was described as an emergent field merging strategies of tissue engineering, stem cell-based therapies, biomaterials, nanotechnology, and biochemistry [12].

The first generation of biomaterials were developed to be used as prosthesis, and to minimize the risk of toxic reactions or rejection they were mainly bioinert. To replace living tissues, bioactive and/or biodegradable biomaterials were developed based on collagen, hydrogel, hydroxyapatite, calcium phosphate, bioglass and glass-ceramics, synthetic resorbable and non-resorbable polymers, and others. Up to now, none of the commercially available biomaterials fulfill the condition of being an ideal bone substitute, and composite and functionalized scaffolds with growth factors, cells, and signaling molecules have been studied and represent a third generation of biomaterials. Cells can be used either combined with biomaterials in tissue engineering procedures or alone in cell therapy. However, many questions related to cell source that still require clarification: whether they are undifferentiated or differentiated cells, whether the cells are manipulated or not, and whether the cells are harvested or genetically edited, among others. Even evidence of the advantages of using cells present contradictory views [13, 14]. To regenerate bone, growth factors have gained attention since the discovery of bone morphogenetic proteins [15] and, although they can work either by stimulating or inhibiting physiologic processes, only growth factors with stimulatory effects have been used, including vascular endothelial growth factor, fibroblast growth factor, and platelet-derived growth factor [16]. Growth factors can be used as cocktails obtained from platelet rich plasma, platelet rich fibrin, platelet poor plasma, and cell lysates where both the composition and concentration of these factors are uncertain. While conceptually promising in the oral surgery field, regenerative medicine would widen its clinical applications with improvements in biomaterial development, knowledge of stem cells, and comprehension and identification of growth factors either alone or in cocktails.

In the last two decades, the incorporation of technologies based on 3D-imaging, virtual surgical planning, and intraoperative navigation revolutionized the way that oral surgery has been carried out. Facial photographs, CT scans, and intraoral scanning images are merged into software to generate a complete virtual study model providing detailed and precise information for diagnosis and surgical planning for trauma, orthognathic and osteogenic distraction surgeries, and oral rehabilitation with dental implants [17]. Modern planning techniques include computer-aided design (CAD)/computer-aided manufacturing (CAM) to fabricate 3D-stereolithographic anatomical models and to customize surgical templates and guides, refining the surgical techniques. Among existing state-of-the-art techniques, bioprinting is a technique still in its infancy, but remains a promising strategy, and has been denominated as a 4D-customized biological scaffold for tissue regeneration.

The advancement of technology has extended beyond planning, and has progressed toward the execution of surgical procedures through intraoperative navigation or dynamic guide-surgery, which could significantly improve surgical precision. Since the development of the da Vinci robotic system, robots have been extensively used for different surgical procedures, with the advantages of preserving underlying structures and improving the outcome with reduced trauma and surgical time [18]. In this context, the trans-oral robotic systems (TORS) have been used to treat some head and neck tumors and obstructive sleep apnea syndrome, as an alternative to conventional open techniques [19, 20]. However, the restricted surgical field and particular anatomy of the maxillofacial area have limited their use in oral surgery. To be routinely employed some challenges have to be overcome, such as the development of specific instruments, the overall cost of the equipment, the need for extensive surgical training, and, most importantly, the transmission of proprioception. The tactile skill of the force applied on screws during a bone fixation, during the implant installation, or even for suture knot-tying, makes humans superior to robots. The use of robotic systems to treat facial traumas and deformities are still limited, mainly due to the lack of mechanical sensitivity needed to properly fix the bone segments [18, 21]. Robots assist on the proper position of implant placement, but their accuracy and validation need to be further investigated [22]. Although promising, there is a lack of scientific evidence to support this technique, and the data are based on surgeon's personal opinions, rather than on scientific reports. Thus, well-designed studies comparing the robotic technique with standard methods could support the safety and feasibility of this technique in the future.

Along with all healthcare surgical procedures, oral surgery is undoubtedly one of humankind's best inventions. From the unknown first procedures to the current ones, there is no question that we have progressed hugely and, by looking ahead, it is very clear that much remains to be done in the years to come. Taking part in this process, the Oral Surgery section of Frontiers in Oral Health will publish all advancements in several areas, looking at either basic, preclinical, or clinical works, and providing a substantial contribution to the field.

## Author Contributions

All authors listed have made a substantial, direct and intellectual contribution to the work, and approved it for publication.

## Conflict of Interest

The authors declare that the research was conducted in the absence of any commercial or financial relationships that could be construed as a potential conflict of interest.
